# Supplementary material for: Siderophore cheating and cheating resistance shape competition for iron in soil and freshwater Pseudomonas communities
Source: Nat Commun. 2017 Sep 4;8:414. doi: 10.1038/s41467-017-00509-4 (PMC5583256; doi:10.1038/s41467-017-00509-4)
Supplement: Supplementary file 1 — Supplementary Information [file 41467_2017_509_MOESM1_ESM.pdf]

SI GUIDE

File Name: Supplementary Information
Description: Supplementary Figures, Supplementary Tables, Supplementary Methods and Supplementary References.

File Name: Peer Review File
Description:

Supplementary Figures

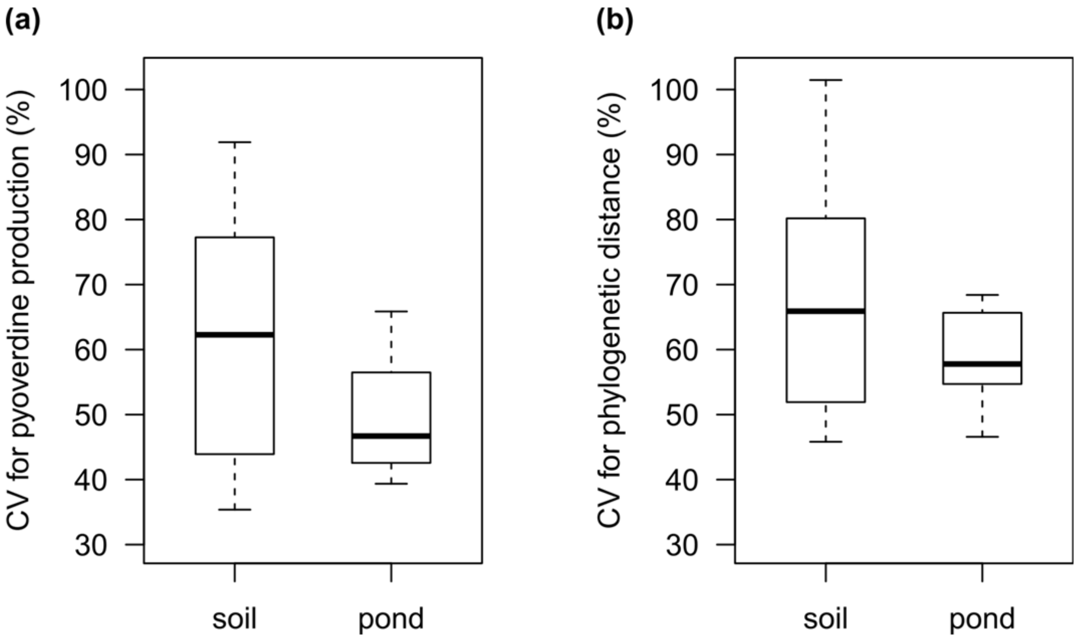

Supplementary Figure 1 | Pyoverdine production and phylogenetic diversity was highly variable in both soil and pond communities. (a) The coefficients of variation (CV = standard deviation / mean) for relative pyoverdine production was high in all communities. **(b)** The CV for phylogenetic distance was also high in both soil and pond communities. For both analyses, the CVs were calculated separately for each of the 16 communities, and based on a total of 158 soil and 157 pond isolates for (a), and 148 soil and 149 pond isolates for (b). Box plots show the median, the 1st and the 3rd quartile, and the 95% confidence interval.

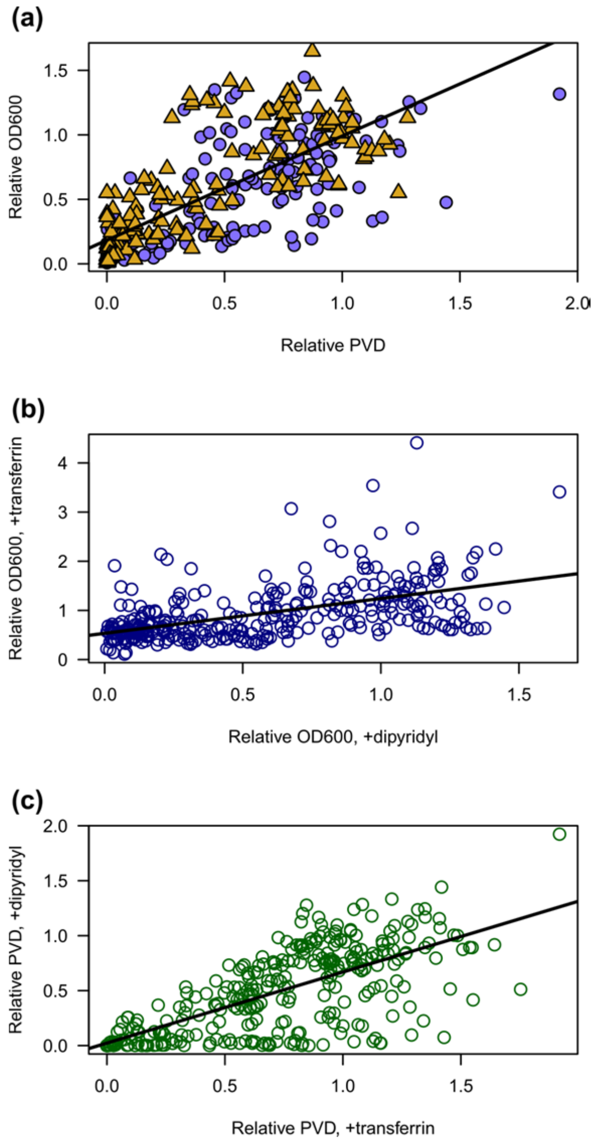

Supplementary Figure 2 | Growth and pyoverdine production of natural isolates significantly correlate between different iron-limited environments. (a) Significant positive correlation between the relative growth (OD_{600}) and the relative pyoverdine production (PVD) of natural soil (yellow triangles, n = 158) and pond (purple circles, n = 157) isolates grown in CAA medium supplemented with the iron chelator 2,2'-dipyridyl (400 µM) (linear mixed model: $t_{298} = 19.67$, $p < 0.001$, solid line). **(b)** Relative growth (OD_{600}) and **(c)** relative pyoverdine production levels for isolates positively correlated between two different iron-limited media (indicated by solid lines): CAA medium with the natural iron chelator apo-transferrin versus CAA medium with the synthetic chelator, 2,2'-dipyridyl (linear models for relative OD_{600}: $t_{313} = 10.6$, $p < 0.001$, $R^2 = 0.262$; relative pyoverdine production: $t_{313} = 16.9$, $p < 0.001$, $R^2 = 0.475$). Values represent means across three replicates.

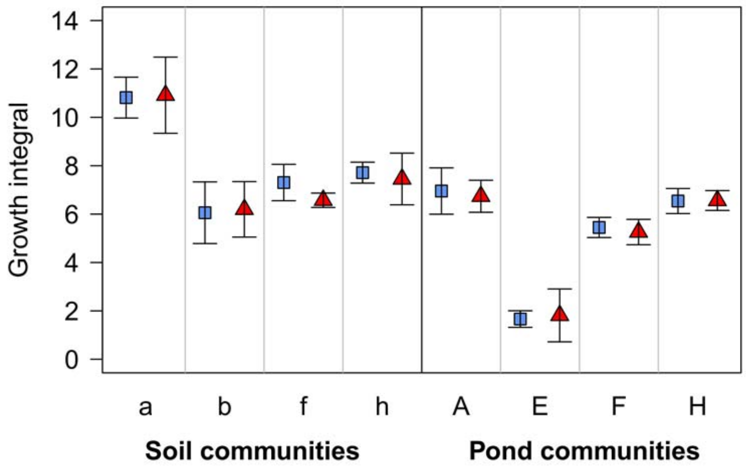

Supplementary Figure 3 | The introduction of a constitutive mCherry marker did not affect strain growth. We fluorescently tagged eight non-producers to be used in direct competition assays against producers. To test whether the fluorescent marker itself has a fitness effect, we grew tagged (red triangles) and untagged (blue squares) non-producers in iron-limited CAA medium as monocultures. There was no significant growth difference between tagged and untagged strains (paired *t*-test: $t_7 = -1.17$, $p = 0.279$). Strains were grown for 48 h as static cultures. Values are given as means ± 95% confidence intervals across three replicates.

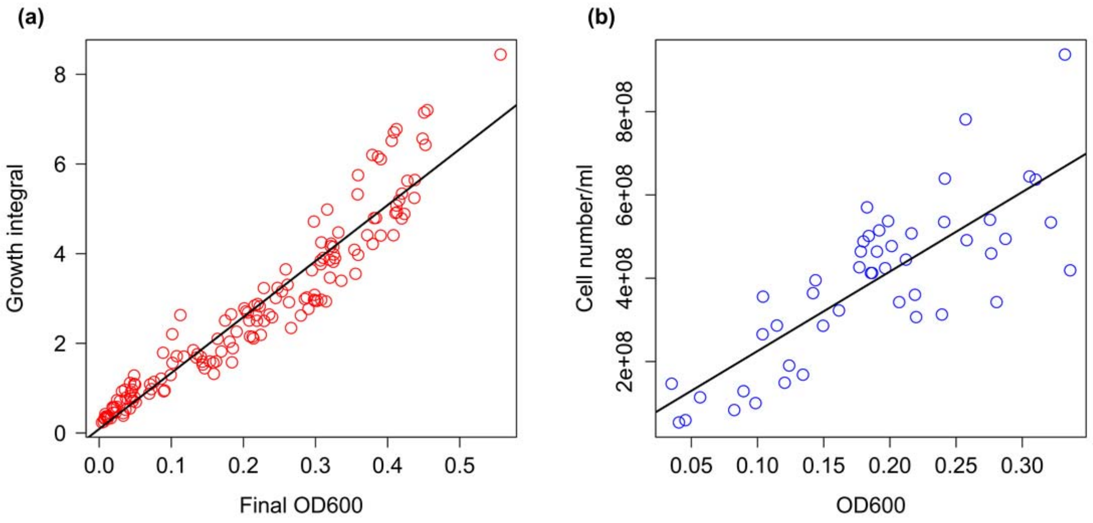

Supplementary Figure 4 | Final OD_{600} is an accurate measure of bacterial growth and cell density. In our high-throughput assays, we used final OD_{600} as a proxy for culture growth. Because we worked with environmental isolates that differ in many aspects, we carried out two control experiments to confirm that final OD_{600} is a reliable measure of growth. **(a)** For a subset of isolates (n = 78 for soil, n = 77 for pond), we compared final OD_{600} to growth integrals obtained from 24-h kinetic growth measurements in iron-limited medium (CAA with 400 µM 2,2'-dipyridyl). We found a strong significant positive correlation between the two growth measurements (linear model: $t_{153} = 45.89$, $p < 0.001$, $R^2 = 0.932$, solid line). Values represent means across three replicates. **(b)** For another subset of isolates (each n = 24 for soil and pond), we compared final OD_{600} to cell count measures (cells/ml) obtained from flow cytometry. We also found a strong positive correlation between the two measurements of growth (linear model: $t_{46} = 9.06$, $p < 0.001$, $R^2 = 0.633$, solid line).

Supplementary Tables

Supplementary Table 1 | Reference strains used in growth and fluorescence measurement assays.

**Strain**	**Description**	**Source or reference**
P. aureofaciens ATCC13985	wildtype	L. Eberl strain collection, University of Zurich
P. entomophila	wildtype	L. Eberl strain collection, University of Zurich
P. protegens CHA0	wildtype	[1]
P. putida IsoF	wildtype, isolated from tomato rhizosphere	[2]
P. syringae B728a	wildtype	L. Eberl strain collection, University of Zurich
P. aeruginosa PAO1 (ATCC15692)	wildtype; pyoverdine type I	[3,4]
P. aeruginosa 2-164	CF isolate United States; pyoverdine type II	[5,6]
P. aeruginosa ATCC 013	Laboratory isolate United States; pyoverdine type III	[5,6]

Supplementary Table 2 | GenBank accession or locus tag numbers of partial or complete *rpoD* sequences used as an outgroup or references for phylogenetic trees.

Strain	*rpoD* accession or locus tag	Group	Subgroup
P. aeruginosa PAO1	NP_249267	*P. aeruginosa*	
P. chlororaphis subsp. aureofaciens 30-84	PCHL3084_RS27440	*P. fluorescens*	*P. chlororaphis*
P. kilonensis 520-20T (DSM 13647T)	AM084336	*P. fluorescens*	*P. corrugata*
P. fluorescens A506	CP003041	*P. fluorescens*	*P. fluorescens*
P. marginalis NCPPB 667	AB039575	*P. fluorescens*	*P. fluorescens*
P. meridiana CIP 108465T	FN554485	*P. fluorescens*	*P. gessardi*
P. jessenii CIP 105274T	FN554473	*P. fluorescens*	*P. jessenii*
P. umsongensis LMG 21317T	FN554516	*P. fluorescens*	*P. jessenii*
P. koreensis LMG 21318T	FN554476	*P. fluorescens*	*P. koreensis*
P. fluorescens R124	I1A 004757	*P. fluorescens*	*P. koreensis*
P. moraviensis DSM 16007T	FN554490	*P. fluorescens*	*P. koreensis*
P. helmanticensis OHA11	HG940517	*P. fluorescens*	*P. koreensis*
P. lini CIP 107460T	FN554478	*P. fluorescens*	*P. mandelii*
P. frederiksbergensis DSM 13022T	AM084335	*P. fluorescens*	*P. mandelii*
P. fluorescens ATCC 17467	AB039530	*P. fluorescens*	
P. graminis LMG 21661T	FN554469	*P. lutea*	
P. putida KT2440	NC_002947	*P. putida*	
P. alkylphenolica JCM 16553T	HE577794	*P. putida*	
P. japonica JCM 21532T	HE577795.	*P. putida*	
P. cichorii NCPPB 943	AB039526	*P. syringae*	
P. syringae pv. tomato DC3000	PSPTO_0537	*P. syringae*	

Supplementary Table 3 | Software packages used for data analysis.

Package	Application	Reference
MEGA 7 software	Phylogenetic analysis	[7]
iTOL web tool	Plotting of phylogenetic trees	[8]
EMBOSS water	Relatedness analysis between pairs of sequences	www.ebi.ac.uk/tools/psa/emboss_water/
grofit	Growth curve analysis in R	[9]
Image J	Image analysis	[10]
Trimmomatic	Filtering of sequence reads	[11]
SPAdes 3.10.1	Assembling sequence reads	[12]
RAST automated annotation pipeline	Identification and annotation of putative coding sequences	[13,14]
OrthoFinder	Identification of gene family profiles	[15]
PKS/NRPS analysis website	Predicting amino acid sequence of non-ribosomal peptide synthetases	[16]
APE v3.2	Phylogenetic analysis in R	[17]
picante v1.6-2	Calculating Blomberg's K-values	[18]

Supplementary Table 4 | Strains used for fluorescent tagging.

Strain	Relevant properties	Source
E. coli S17-1 λpir pUX-BF13	with conjugation elements and helper plasmid	L. Eberl strain collection, University of Zurich
E. coli S17 λpir miniTn7-Ptac-mCherry	with mini-Tn7 plasmid carrying mCherry under constitutive promoter	J. van der Meer, University of Lausanne
E. coli S17-1 λpir miniTn7-Ptac-mCherry	with conjugation elements and mini-Tn7 plasmid carrying mCherry under constitutive promoter	L. Eberl strain collection, University of Zurich

Supplementary Methods

Pyoverdine purification assay. We adapted the method of Meyer et al. [19] to crudely purify pyoverdine from the 16 producer strains. Briefly, we added 2 ml of producer overnight LB culture to 500 ml CAA with 200 µM 2,2'-dipyridyl in a 1 L glass flask, and let cultures grow for 24 h at 25°C shaken (100 rpm). Afterwards, we centrifuged cultures at 7,500 rcf for 15 min (in 50 ml aliquots). We acidified supernatants with HCl 1 M till pH = 6 and centrifuged again at 5,000 rcf for 10 min. The supernatant was then added on a XAD-4 (Amberlite) column with a flux of two drops per second. The column was washed with 300 ml of Milli-Q water. 50% methanol (in Milli-Q water) was subsequently used as an eluent. We collected fractions of the eluate that showed peak pyoverdine fluorescence (150 – 250 ml). The fractions were distributed in Petri dishes and left for 24 h under a hood to let methanol evaporate. The residues were first dissolved in Milli-Q water, then combined and lyophilised for 48 h (Lyovac). Columns were regenerated by washing with 1 L of methanol containing 1% of concentrated HCl (32%) and then washed with 1 L of Milli-Q water.

Fluorescent tagging. We tagged the eight non-producers with a red fluorescent mCherry protein gene via electroporation or conjugation using a mini-Tn7 system for chromosomal integration [20]. Electroporation protocol was adapted from Choi & Schweizer [21]. We used donor strains *Escherichia coli* S17 λpir and S17-1 λpir carrying a plasmid with the mini Tn7-mCherry construct under constitutive promoter [22], and a helper strain *E.coli* S17-1 λpir carrying a helper plasmid (pUX-BF13) [23]. S17-1 strains additionally contain a chromosomal insertion with the conjugation elements and they were used for conjugation.

Electroporation. *E. coli* donor and helper strains were grown in 4 ml of LB supplemented with an appropriate antibiotic in 14 ml polypropylene round-bottom tubes shaken (200 rpm)

at 37 °C for 24 h. Plasmids were purified using a ZR Plasmid Miniprep-Classic kit (Zymo Research) following manufacturer's instructions. Main modifications to the protocol of Choi & Schweizer [21]: (a) we harvested isolates grown in LB at OD_{600} = 0.3 - 0.7; (b) 500 ng - 1.8 µg of each plasmid were used; (c) after electroporation we recovered bacteria for 3 - 4.5 h at 28 °C shanking (160 rpm), and then (d) plated them on LB-agar (12%) with different gentamycin concentration (8, 30, 35 or 45 µg/ml), and incubated the plates at room temperature for 2 - 3 days.

Conjugation. We pelleted overnight cultures (grown as described above or in 5 ml of LB in 50 ml falcon tubes) of donor, helper, and recipient (soil or pond non-producer) at 7,500 rcf for 5 min. Then we washed the pellets with 2 ml of a 0.85% NaCl solution, pelleted at the same speed and suspended the pellets in LB broth, so that the donor and helper were 2 - 4 times more concentrated than the recipient. A mixture of the three bacteria was incubated at 28 °C overnight as two 50 µl drops on an LB-agar (12%) plate. Afterwards, we suspended the drops in 800 µl of 0.85% NaCl solution, and spread 10 µl, 100 µl and the remaining concentrated culture on *Pseudomonas* selective plates (PIA, *Pseudomonas* isolation agar) with a proper gentamycin concentration (8, 30 or 45 µg/ml). Plates were incubated at room temperature for 2 - 4 days.

Three single colonies per isolate that were fluorescing when checked with Infinity3 camera system (Lumenera corporation), were streaked out for single colonies on LB-agar plates. We wanted to choose those transformants that grew most similar to their untagged version. For this, we first pre-grew the untagged and tagged strains in LB and then in CAA with 200 µM 2,2'-dipyridyl and compared their growth (OD_{600} after 24 h of incubation at room

temperature; measured with Tecan microplate reader). The tagged strains that grew most similar to their wildtype were chosen for the next experiments.

We further wanted to check whether mCherry marker has fitness consequences for the chosen transformants (Supplementary Fig. 3). For this, we first grew untagged and tagged versions of the non-producers in LB overnight. Then we adjusted cultures to $OD_{600} = 1$ with LB and added 2 µl of the adjusted cultures to 200 µl of CAA with 200 µM 2,2'-dipyridyl in 96-well plates, in triplicates. OD_{600} was measured every 30 min at room temperature (25 - 28 °C) for 48 h in a Tecan microplate reader. Growth curves were analysed as described in the section 'Measurement of growth and pyoverdine production levels'. Statistical analysis revealed that, overall, mCherry marker did not impair growth of the isolates (Supplementary Fig. 3).

Supplementary References

1. Laville, J. *et al.* Characterization of the *hcnABC* gene cluster encoding hydrogen cyanide synthase and anaerobic regulation by ANR in the strictly aerobic biocontrol agent *Pseudomonas fluorescens* CHA0. *J. Bacteriol.* **180**, 3187-3196 (1998).
2. Steidle, A. *et al.* Identification and characterization of an N-acylhomoserine lactone-dependent quorum-sensing system in *Pseudomonas putida* strain IsoF. *Appl. Environ. Microbiol.* **68**, 6371-6382 (2002).
3. Stover, C. K. *et al.* Complete genome sequence of *Pseudomonas aeruginosa* PAO1, an opportunistic pathogen. *Nature* **406**, 959-964 (2000).
4. Ghysels, B. *et al.* FpvB, an alternative type I ferripyoverdine receptor of *Pseudomonas aeruginosa*. *Microbiology* **150**, 1671-1680 (2004).
5. Smith, E. E., Sims, E. H., Spencer, D. H., Kaul, R. & Olson, M. V. Evidence for diversifying selection at the pyoverdine locus of *Pseudomonas aeruginosa*. *J. Bacteriol.* **187**, 2138-2147 (2005).
6. Jiricny, N. *et al.* Fitness correlates with the extent of cheating in a bacterium *J. Evol. Biol.* **23**, 738-747 (2010).
7. Kumar, S., Stecher, G. & Tamura, K. MEGA7: molecular evolutionary genetics analysis version 7.0 for bigger datasets. *Mol. Biol. Evol.* **33**, 1870-1874 (2016).
8. Letunic, I. & Bork, P. Interactive Tree Of Life v2: online annotation and display of phylogenetic trees made easy. *Nucleic Acids Res.* **39**, W475-478 (2011).
9. Kahm, M., Hasenbrink, G., Lichtenberg-Fraté, H., Ludwig, J. & Kschischo, M. grofit: fitting biological growth curves with R. *J. Stat. Softw.* **33** (2010).
10. Schneider, C. A., Rasband, W. S. & Eliceiri, K. W. NIH Image to ImageJ: 25 years of image analysis. *Nat. Methods* **9**, 671-675 (2012).
11. Bolger, A. M., Lohse, M. & Usadel, B. Trimmomatic: a flexible trimmer for Illumina sequence data. *Bioinformatics* **30**, 2114-2120 (2014).
12. Bankevich, A. *et al.* SPAdes: a new genome assembly algorithm and its applications to single-cell sequencing. *J. Comput. Biol.* **19**, 455-477 (2012).
13. Aziz, R. K. *et al.* The RAST Server: rapid annotations using subsystems technology. *BMC Genomics* **9**, 75 (2008).
14. Overbeek, R. *et al.* The SEED and the Rapid Annotation of microbial genomes using Subsystems Technology (RAST). *Nucleic Acids Res.* **42**, D206-214 (2014).

15. Emms, D. M. & Kelly, S. OrthoFinder: solving fundamental biases in whole genome comparisons dramatically improves orthogroup inference accuracy. *Genome Biology* **16**, 157 (2015).
16. Bachmann, B. O. & Ravel, J. Methods for *in silico* prediction of microbial polyketide and nonribosomal peptide biosynthetic pathways from DNA sequence data. *Methods Enzymol.* **458**, 181-217 (2009).
17. Paradis, E., Claude, J. & Strimmer, K. APE: analyses of phylogenetics and evolution in R language. *Bioinformatics* **20**, 289-290 (2004).
18. Kembel, S. W. *et al.* Picante: R tools for integrating phylogenies and ecology. *Bioinformatics* **26**, 1463-1464 (2010).
19. Meyer, J. M. *et al.* Use of siderophores to type pseudomonads: the three *Pseudomonas aeruginosa* pyoverdine systems. *Microbiology* **143**, 35-43 (1997).
20. Lambertsen, L., Sternberg, C. & Molin, S. Mini-Tn7 transposons for site-specific tagging of bacteria with fluorescent proteins. *Environ. Microbiol.* **6**, 726-732 (2004).
21. Choi, K.-H. & Schweizer, H. P. mini-Tn7 insertion in bacteria with single attTn7 sites: example *Pseudomonas aeruginosa*. *Nat. Protoc.* **1**, 153-161 (2006).
22. Rochat, L., Péchy-Tarr, M., Baehler, E., Maurhofer, M. & Keel, C. Combination of fluorescent reporters for simultaneous monitoring of root colonization and antifungal gene expression by a biocontrol pseudomonad on cereals with flow cytometry. *Mol. Plant Microbe Interact.* **23**, 949-961 (2010).
23. Bao, Y., Lies, D. P., Fu, H. & Roberts, G. P. An improved Tn7-based system for the single-copy insertion of cloned genes into chromosomes of Gram-negative bacteria. *Gene* **109**, 167-168 (1991).
